# Vaccine Adjuvants in Fish Vaccines Make a Difference: Comparing Three Adjuvants (Montanide ISA763A Oil, CpG/Poly I:C Combo and VHSV Glycoprotein) Alone or in Combination Formulated with an Inactivated Whole Salmonid Alphavirus Antigen

**DOI:** 10.3390/vaccines2020228

**Published:** 2014-03-25

**Authors:** Hanna L. Thim, Stéphane Villoing, Marian McLoughlin, Karen Elina Christie, Søren Grove, Petter Frost, Jorunn B. Jørgensen

**Affiliations:** 1 Norwegian College of Fisheries Science, UiT The Arctic University of Norway, Tromsø N-9037, Norway; E-Mail: hanna.l.thim@uit.no; 2 MSD Animal Health Norway, Thormøhlensgate 55, Bergen N-5008, Norway; E-Mails: stephane.villoing@merck.com (S.V.); karenelina.christie@merck.com (K.E.C.); petter.frost@merck.com (P.F.); 3 Aquatic Veterinary Services, 35 Cherryvalley Park, Belfast BT5 6PN, UK; E-Mail: marian@aquatic-veterinary.co.uk; 4 Department of Laboratory Services, National Veterinary Institute, Oslo N-0106, Norway; E-Mail: soren.grove@vetinst.no

**Keywords:** salmonid alphavirus, VHSV, vaccination, adjuvant, DNA vaccine, CpG, polyI:C, complement, neutralizing antibodies

## Abstract

Most commercial vaccines offered to the aquaculture industry include inactivated antigens (Ag) formulated in oil adjuvants. Safety concerns are related to the use of oil adjuvants in multivalent vaccines for fish, since adverse side effects (e.g., adhesions) can appear. Therefore, there is a request for vaccine formulations for which protection will be maintained or improved, while the risk of side effects is reduced. Here, by using an inactivated salmonid alphavirus (SAV) as the test Ag, the combined use of two Toll-like receptor (TLR) ligand adjuvants, CpG oligonucleotides (ODNs) and poly I:C, as well as a genetic adjuvant consisting of a DNA plasmid vector expressing the viral haemorrhagic septicaemia virus (VHSV) glycoprotein (G) was explored. VHSV-G DNA vaccine was intramuscularly injected in combination with intraperitoneal injection of either SAV Ag alone or combined with the oil adjuvant, Montanide ISA763, or the CpG/polyI:C combo. Adjuvant formulations were evaluated for their ability to boost immune responses and induce protection against SAV in Atlantic salmon, following cohabitation challenge. It was observed that CpG/polyI:C-based formulations generated the highest neutralizing antibody titres (nAbs) before challenge, which endured post challenge. nAb responses for VHSV G-DNA- and oil-adjuvanted formulations were marginal compared to the CpG/poly I:C treatment. Interestingly, heat-inactivated sera showed reduced nAb titres compared to their non-heated counterparts, which suggests a role of complement-mediated neutralization against SAV. Consistently elevated levels of innate antiviral immune genes in the CpG/polyI:C injected groups suggested a role of IFN-mediated responses. Co-delivery of the VHSV-G DNA construct with either CpG/polyI:C or oil-adjuvanted SAV vaccine generated higher CD4 responses in head kidney at 48 h compared to injection of this vector or SAV Ag alone. The results demonstrate that a combination of pattern recognizing receptor (PRR) ligands, such as CpG/polyI:C, increases both adaptive and innate responses and represents a promising adjuvant strategy for enhancing the protection of future viral vaccines.

## 1. Introduction

Viral diseases present a huge problem for the global aquaculture industry, where infectious diseases are estimated to be responsible for a production loss of ten to twenty percent each year [1]. Among these, pancreas disease (PD) caused by salmon pancreas disease virus, now more commonly referred to as salmonid alphavirus (SAV), is responsible for big economic losses throughout the Norwegian, Scottish and Irish aquaculture industry.

SAV strains are grouped into six different subtypes (SAV1-6), based on sequencing and phylogenetic analysis [2]. SAV1 isolates are found in Ireland and the U.K., while SAV3 isolates are exclusively found in Norway and affects both Atlantic salmon and rainbow trout [3,4]. PD histopathological signs are characterized by lesions in pancreatic acinar tissue, heart and, later, also in skeletal muscle [5,6]. Several studies have demonstrated protective immune responses against SAV in salmonids, both experimentally and in the field [7,8], and the protection has been shown to be associated with antibody (Ab)-mediated immunity and neutralizing Ab (nAbs) [7,9,10]. Following passive immunization of salmon parr and post smolts with SAV antisera, the fish are reported to be protected upon re-challenge, which indicates that protective immunity is conferred by NAbs [11]. Since then, various vaccination strategies against SAV have been tested both in Atlantic salmon and in rainbow trout, such as an inactivated SAV vaccine based on a Subtype 1 isolate [12], a recombinant live attenuated SAV vaccine (Subtype 2 isolate) [13] and subunit and DNA vaccines based on an SAV Subtype 3 isolate [14]. An inactivated whole-virus vaccine based on an Irish SAV Subtype 1 isolate has been used in Norway, Ireland and the U.K. since 2007 [15].

Most of the commercial finfish vaccines against viruses are, like the above-mentioned SAV vaccine, based on inactivated antigens (Ag) or recombinant subunit proteins formulated in oil emulsions. Oil-based adjuvants are based on creating a depot of Ag, which improves Ag delivery to Ag-presenting cells (APC) or by attracting effector cells to the site of injection. However, side effects due to oil adjuvants have been reported and are expressed both physiologically and morphologically [16,17,18]. Administration of these oil-based vaccines is performed by intraperitoneal (i.p.) injection, and relatively high doses are needed to achieve protection [19]. Hence, more potent adjuvants enabling the use of a lower Ag dose and reducing side effects would advantageously be used for fish vaccines. Many adjuvants are derived from pathogens and act via cell-associated germ line-encoded pattern recognition receptors (PRR) on APCs to provide a danger signal. Such adjuvants induce maturation of the APCs and enhance Ag presentation and associated co-stimulation [20]. Among the best studied PRRs is the Toll-like receptor (TLR) family, of which 17 types have been described in different fish species [21]. The piscine TLRs includes TLR9 recognizing bacterial and viral DNA [22,23] and TLR3 and TLR22, which both recognize double-stranded (ds) RNA [24,25]. Studies by us and other groups have demonstrated that ligands for these receptors, such as synthetic CpG oligonucleotides (ODNs; TLR9 ligand) and polyI:C (TLR3/22 ligand) can stimulate the production of pro-inflammatory cytokines/chemokines and Type I IFNs, which increase the host’s ability to eliminate viral pathogens [26,27]. Further, our group has, in accordance with mammalian studies [28], shown that a combined treatment of CpG/polyI:C induces synergistic upregulation of a wide array of immune genes in Atlantic salmon [26] and significantly enhances protection on its own against SAV [29]. When formulated in an SAV whole-virus Ag formulation [30], the combo significantly increased antibody-mediated clearance of SAV from blood, thus preventing the development of SAV-specific heart lesions. This strongly indicates that a humoral Ab response is important for protection against SAV and that CpG/polyI:C boost this protection.

The Novirhabdovirus VHSV (viral haemorrhagic septicaemia virus) glycoprotein (G) DNA-vaccine is an intra-muscularly (i.m.) injected vaccine that has been shown to induce early non-specific, as well as long-lasting specific protection against VHSV in trout [31,32] and seems to act as a genetic adjuvant [33]. Several studies have suggested that the effects of DNA-vaccines are regulated by innate responses through PRR-signalling cascades [34], and although the mechanisms that induce early protective responses by the VHSV-G DNA construct are still unclear, innate signalling are most likely involved. The VHSV-G DNA vaccine has been shown to create a local immune-competent environment at the i.m. injection site [35], and an interesting question addressed here was whether i.m. co-injection of this DNA vaccine simultaneously with an i.p. administered TLR-agonist-adjuvanted vaccine based on an inactivated SAV Ag could provide improved protective responses. Combining several PRR agonists has previously shown synergistic effects when intended as adjuvants, resulting in enhanced and more durable responses to Ag, as well as dose sparing effects [36,37]. To our knowledge, this is the first report of immunity associated with inactivated virus Ag vaccines formulated with mixed TLR agonists and with VHSV-G (vhsG) DNA vaccine as a genetic adjuvant. In the current study, the protective effects between an i.p. injection of SAV Ag with CpG/polyI:C were compared to the same treatment combined with a simultaneous i.m. injection of the vhsG DNA vaccine to see whether additive or synergistic effects were induced. To evaluate vhsG adjuvant effects, several controls were included, whose effects have been investigated earlier [29,30]; SAV Ag was i.p. injected alone or with an oil adjuvant (Montanide ISA763A), formulations of which were co-injected i.m. with vhsG to thoroughly examine the possible effects these adjuvant combinations could have on protection and immune responses against an SAV Subtype 3 challenge. 

## 2. Experimental

### 2.1. Reagents and Constructs

DNA plasmid pcDNA3-vhsG [38] was kindly provided by Dr. Niels Lorenzen and diluted in 1× PBS to a concentration of 0.2 mg/mL. The synthetic dsRNA (poly I:C; Merck, Nottingham, UK) and the phosphorothioate-modified class B CpG oligonucleotide (2006T: TCGTCGTTTTGTCGTTGTCGTT, Thermo Scientific, Ulm, Germany) were dissolved in TE buffer (10 mM Tris, 1 nM EDTA, pH 8) at 5 mg/mL and further diluted 10-fold in the final vaccine formulations (50 µg/dose). SAV Ag was prepared by propagating the SPDV/SAV strain F93-125 in cell culture, which then was formalin inactivated. To demonstrate the potential additive or synergistic effects of the adjuvants tested, the same suboptimal dose of inactivated SAV Ag was used for all formulations. Montanide ISA 763 (Seppic, France) was used to prepare the water-in-oil formulations, by dispersing the water phase (containing the formalin inactivated SAV Ag) into the vegetable oil phase (containing emulsifiers and stabilizers) and emulsified using a homogenizer with an emulsification rotor. All SAV Ag formulations were provided by MSD Animal Health (Bergen, Norway). 

### 2.2. Fish

The experimental challenge study was performed at ILAB’s challenge lab facility at the University of Bergen (Høyteknologisenteret, Bergen, Norway), which fulfil the confinement conditions required for working with GMOs and DNA vaccines. Atlantic salmon, pre-smolt (Fister) with a mean weight of approximately 29 g at time of vaccination were kept in tanks supplied with running fresh water at 11–12 °C and fed with commercial dry feed (Skretting, Stavanger, Norway) based on appetite. The fish were starved for a minimum of 48 h and anaesthetized with metacainum (0.1 mg/mL bath treatment) prior to all handling.

### 2.3. Vaccination and SAV Cohabitation Challenge

Fish were divided into 7 treatment groups (*n* = 65, 69 or 75, depending on the required sampling size) and a saline injected control group (*n* = 79). As described in detail in Table 1, three treatment groups were i.p. injected with 100 μL of the SAV1 whole-inactivated virus Ag formulation, formulated with or without oil and/or 50 µg CpG/polyI:C. Moreover, three treatment groups were, in parallel to the i.p. injections, injected i.m. with 10 µg of the PcDNA3-vhsG plasmid diluted in 50 µL PBS (1×). One group received PcDNA3-vhsG plasmid alone, and the control group was injected with 100 μL of PBS. Fish were marked by fin and/or maxilla clipping, and there were no mortalities observed after injection. A total of 570 fish were used and divided into 3 tanks; 1 tank for the SAV cohabitation challenge (422 fish excluding shedders), and 2 tanks for harvesting organs to monitor early immune gene expression (2 × 74 fish). At 6 weeks post vaccination (wpv), 86 Atlantic salmon were injected i.p. with 0.2 mL SAV Subtype 3, each receiving a viral dose of about 1 × 10^3^ TCID_50_ and added to the SAV challenge tank to serve as shedders (*n* = 86 corresponds to 20% of the final amount of fish in the tank). The shedders were marked with a red VIE label under the anal fin one week prior to challenge.

**Table 1:** Treatments, dose regime, number of fish and schedule for the sampling of organs and blood.

Treatment	Total # of fish	Analysis (# of fish)					
		Immune gene expression 12 and 48 hpv	nsP1 RT-qPCR 3 wpc ^1^	Histopathology 5 and 6 wpc ^2^	nAb assay		
					6 wpv	3 wpc ^1^	6wpc ^2^
**SAV Ag**	74 + 1	8 + 8	10	15 + 15	13	15	**15**
**SAV Ag oil**	64 + 1	8 + 8	10	15 + 15	8	10	**15**
**SAV Ag CpG (50 µg)/poly I:C (50 µg)**	74 + 1	8 + 8	10	15 + 15	13	15	**15**
**SAV Ag PcDNA3-vhsG (10 µg)**	68 + 1	10 + 10	10	15 + 15	8	10	**15**
**SAV Ag oilPcDNA3-vhsG (10 µg)**	68 + 1	10 + 10	10	15 + 15	8	10	**15**
**SAV Ag CpG (50µg)/poly I:C (50 µg) PcDNA3-vhsG (10 µg)**	68 + 1	10 + 10	10	15 + 15	8	10	**15**
**PcDNA3-vhsG (10 µg)**	68 + 1	10 + 10	10	15 + 15	8	10	**15**
**Saline 0.9%**	**78 + 1**	**10 + 10**	**10**	**15 + 15**	**13**	**15**	**15**

The treatment that is underlined, PcDNA3-vhsG, was intra-muscularly (i.m.) injected parallel to intraperitoneal (i.p.) injection of SAV Ag treatments. Five extra fish were sampled at 6 wpv and 3 wpc for analyses not included here. hpv; hours post vaccination; wpc; weeks post challenge. ^1^ The same fish sampled for nsP1 RT-qPCR as for the nAb assay; ^2^ the same fish were sampled for heart histopathology and for the nAb assay. SAV, salmonid alphavirus; Ag, antigen; nAb, neutralizing antibody.

### 2.4. RT-qPCR of Immune Gene Expression: RNA Isolation, cDNA Synthesis and RT-qPCR

Spleen and head kidney (HK) were harvested from 8 fish per group at 12 and 48 h post vaccination (hpv) and were stored according to the manufacturer’s guidelines on RNAlater (Ambion, Applied Biosystems, Foster City, CA, USA). RNA isolation, cDNA synthesis and RT-qPCR were executed as described previously [26] with minor changes. Four hundred nanograms of total RNA were reverse transcribed (TaqMan Reverse Transcription Reagents kit; Applied Biosystems) into cDNA using random hexamer primers in 30-μL reaction volumes following the manufacturer’s guidelines. Primer and probe sequences and the efficiencies of the assays used in this study are presented in Table 2. cDNA samples (2.5 µL) were analysed in duplicates (target genes) or triplicates (endogenous control) in 20-µL reactions on a 7500 Fast Real-Time PCR system. The Cq-threshold was automatically set to 0.2 for analysis of both endogenous and target genes. Relative expression and statistics were calculated using Relative Expression Software Tool (REST) 2009 [39], which is based on Pfaffl’s mathematical model [40], where individual Cq-values were compared between saline-injected fish (control) and vaccine injected fish (test) and correlated to the endogenous control gene, EF1αβ, and PCR-efficiency. 

### 2.5. SAV Neutralizing Ab Responses

Serum samples from 8 to 15 individuals per group and time point (see Table 1 for details) were collected at 6 wpv and 3 and 6 wpc (weeks post challenge) and examined for SAV-neutralizing activity. To do so, virus initially incubated with diluted sera was left to adhere to Chinook salmon embryo-214 (CHSE) cells, and after 8 days, the presence of cell-associated virus was detected by an ELISA-method, as reported earlier [30]. Individual sera from each group were pooled, and half of the pooled sera were heat inactivated (HI; 56 °C for 30 min). Assays for both HI and not heat inactivated (NHI) sera were repeated 3 times for all samples. Two-fold dilutions of either HI or NHI serum were added in duplicate to a 96-well microtiter plate with maintenance media (MM; Minimum essential medium eagle (MEM) supplemented with 2% Foetal bovine serum (FBS)), giving a final dilution range from 1:20 to 1:640 (1:160 to 1:5,120 for the CpG/polyI:C-treated groups) when 100 µL of virus supernatant SPDV (SAV Subtype 1 isolate, 6,000 TCID_50_/mL) were added to the wells containing salmon serum dilutions. Neutralizing effects in serum were expressed as the highest reciprocal titres showing a >50% reduction of the positive control OD value using the following formula:

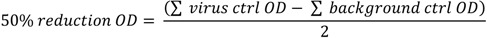



### 2.6. SAV nsP1 RT-qPCR Detection

To measure SAV levels during the viraemic phase, a quantitative real-time RT-PCR (RT-qPCR) was performed on viral RNA extracted from sera 3 wpc from 10 individuals per group, as described previously [26]. Non-structural protein 1 (nsP1) primers and the probe used for this assay are described in Table 1. Individual Cq-values were transformed to relative numbers by the following formula, where *y* represents the lowest Cq-value detected (*i.e*., the highest number of nsP1 transcripts) and where *x* is any of the other Cq-values detected:


RelCq(*x*) = 2^(y-x)^

A sample was considered infected when it had a relative value between 1.0 × 10^0^ and the cut off value of 3.0 × 10^−7^ (*x* = 37.5).

### 2.7. Histopathology

Heart samples for detecting SAV-induced lesions were collected from 15 fish/group at 5 and 6 weeks post-challenge and immediately fixed in 3.5% formaldehyde in buffered saline at pH 7.0 (4.0 g NaH_2_PO_4_·_2_H_2_O, 6.5 g Na_2_HPO_4_·_2_H_2_O, 100 mL 35% formaldehyde and 900 mL dH_2_O). To evaluate the severity of SAV-induced heart lesions, a previously defined scoring system was used (no lesion: 0; minimal: 1; mild: 2; moderate: 3; severe: 4), where scores of 2 and more are defined to be specifically induced by an SAV infection [41]. The lesion scoring was done by Marian McLoughlin (Aquatic Veterinary Services, Belfast, Ireland) using “blinded” heart samples.

### 2.8. Data Analyses

All analyses were done in GraphPad Prism 5.0 if not mentioned otherwise. Differences in protection (SAV nsP1 RT-qPCR and histology) were statistically evaluated by the Kruskal-Wallis rank sum test with *p* < 0.05 as the significance limit, followed by Dunn’s post hoc test at a 5% level of significance. The histology test parameter used for statistical analysis was the severity of heart lesions, scored on the ordinal scale (0–4). Statistical analysis of the SAV nsP1 RT-qPCR used the individual Cq-values of each group as the test parameters. A modified expression of the relative percent protection score (RPPsc.) [29] was used to evaluate the level of protection against SAV induced by the tested treatments, based on the results obtained with the experimental methods (SAV-induced heart lesions histology or SAV-specific RT-qPCR assay). The advantage with this modified RPPsc. method is that the actual differences in degrees of severity of disease between the affected animals for the treated and control groups are taken into consideration. RT-qPCR data were statistically analysed by the Relative Expression Software Tool (REST 2009 v.2.0.13) [39].

**Table 2:** Primers and probe sequences for quantitative reverse-transcriptase PCR and PCR efficiency.

GENES	ASSAY	PRIMERS/PROBE	SEQUENCE (5'–3')	PCR EFFICIENCY	ACCESSION NO.
**CD4-1**	Fw/Rev 200 nM Probe 200 nM	Forward Reverse Probe	GAATCTGCCGCTGCAAAGAC AGGGATTCCGGTCTGTATGATATCT [6FAM]CCCAAACCAAAAGGATTC[BHQ1]	1.78	EU409792
**CD4-2a**	SYBR® Green 3.75 µM	Forward Reverse	TGCAAAGAAGGCGCAGAT GAAAACCTTTAATTTAACAGG	1.76	EU409793
**CD8a**	Fw/Rev 450 nM Probe 250 nM	Forward Reverse Probe	CGTCTACAGCTGTGCATCAATCAA GGCTGTGGTCATTGGTGTAGTC [6FAM]CTGGGCCAGCCCCTAC[BHQ1]	1.74	AY693391
**EF1aB**	Fw/Rev 900 nM Probe 250 nM SYBR Green* 3.75 µM	Forward * Reverse * Probe	TGCCCCTCCAGGATGTCTAC CACGGCCCACAGGTACTG [6FAM]AAATCGGCGGTATTGG[BHQ1]	2.09/2.16 *	BG933897
**IFNa1**	SYBR Green 3.75 µM	Forward Reverse	CCTTTCCCTGCTGGACCA TGTCTGTAAAGGGATGTTGGGAAAA	2.0	AY2169594 AY2169595
**IFNγ**	Fw/Rev 900 nM Probe 250 nM	Forward Reverse Probe	AAGGGCTGTGATG TGTTTCTGTGTACTGAGCGGCATTACTCC [6FAM]TTGATGGGCTGGATGACTTTAGGA[BHQ1]	2.0	AY795563
**mIgM**	Fw/Rev 200 nM Probe 200 nM	Forward Reverse Probe	CCTACAAGAGGGAGACCGA GATGAAGGTGAAGGCTGTTTT [6FAM]TGACTGACTGTCCATGCAGCAACACC[BHQ1]	2.0	
**Mx1/2**	Fw/Rev 900 nM Probe 250 nM	Forward Reverse Probe	GATGCTGCACCTCAAGTCCTATTA CGGATCACCATGGGAATCTGA [6FAM]CAGGATATCCAGTCAACGTT[BHQ1]	1.96	U66475/U66476
**PAX5**	Fw/Rev 200 nM Probe 200 nM	Forward Reverse Probe	CCACTGCCAGGTCGA GAGTCAGCGAGGAGGTGGAGTA [6FAM]CCCCGGCTATCCACCACACG[BHQ1]	1.85	
**sIgM**	Fw/Rev 900 nM Probe 250 nM	Forward Reverse Probe	CTACAAGAGGGAGACCGGAG AGGGTCACCGTATTATCACTAGTTT TCCACAGCGTCCATCTGTCTTTC	1.94	BT060420
**Vig-1**	Fw/Rev 900 nM Probe 250 nM	Forward Reverse Probe	AGCAATGGCAGCATGATCAG TGGTTGGTGTCCTCGTCAAAG [6FAM]AGTGGTTCCAAACGTATGGCGAATACCTG[BHQ1]	1.94	BT047610
**Q_nsP1**	Fw/Rev 900 nM Probe 130 nM	Forward Reverse Probe	CCGGCCCTGAACCAGTT GTAGCCAAGTGGGAGAAAGCT [6FAM]CTGGCCACCACTTCGA[BHQ1]	-	AY604235

Fw; forward. Rev; reverse. * The same primers were used for SYBR Green as for TaqMan.

## 3. Results

### 3.1. Immune Genes

**Figure 1:**
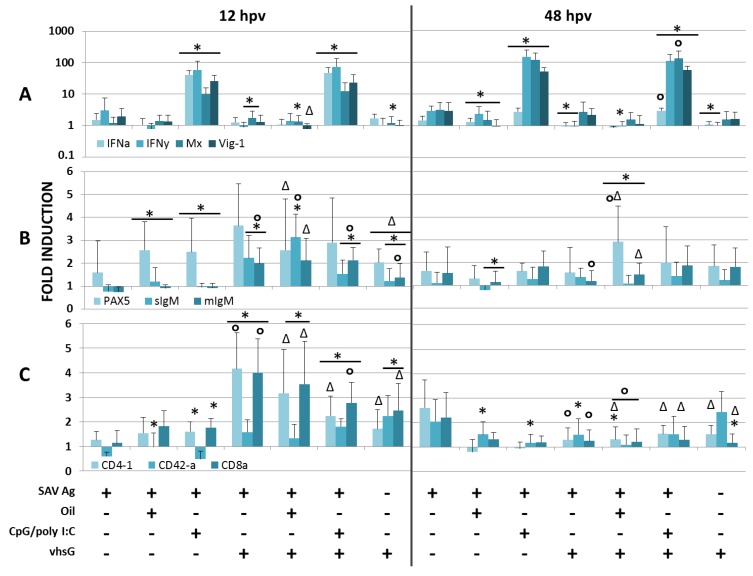
Relative expression of (**A**) antiviral, (**B**) B- and (**C**) T-cell markers in head kidney at 12 and 48 hpv for all treatments compared to saline-treated fish (expression is normalized to reference gene EF1aB). Relative expression is presented as histograms (the colour codes for each gene analysed are indicated below the histograms) calculated from fold induction by Pfaffl’s method (see Materials and Methods), and significant up- or down-regulation is based on data from REST 2009. Significant differences for all treatments compared to SAV Ag are highlighted with an *, against SAV Ag vhsG as Δ and against SAV Ag CpG/polyI:C as °, + or −, respectively, indicates the presence or absence of either SAV Ag, oil, CpG/polyI:C or vhsG.

**Figure 2:**
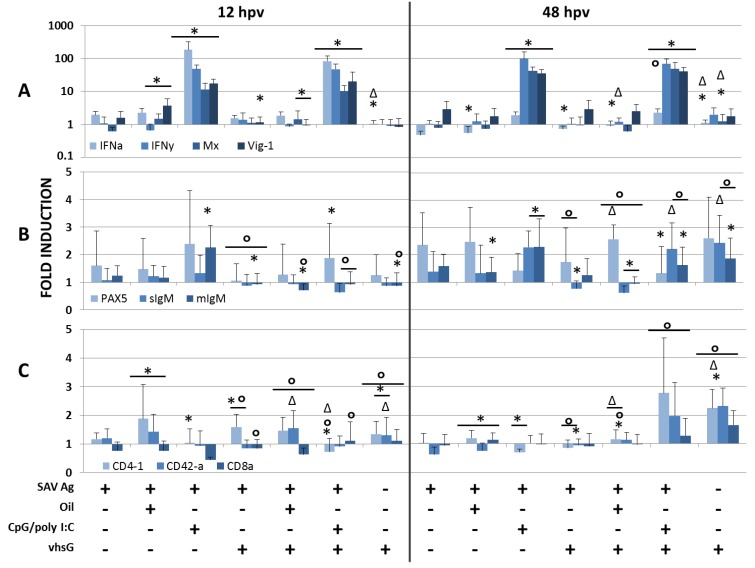
Relative expression of (**A**) antiviral, (**B**) B- and (**C**) T-cell markers in spleen at 12 and 48 hpv for all treatments compared to saline-treated fish (expression is normalized to reference gene EF1aB). Relative expression is presented as histograms (the colour codes for each gene analysed are indicated below the histograms) calculated from fold induction by Pfaffl’s method (see Materials and Methods), and significant up- or down-regulation is based on data from REST 2009. Significant differences for all treatments compared to SAV Ag are highlighted with an *, against SAV Ag vhsG as Δ and against SAV Ag CpG/polyI:C as °, + or –, respectively, indicates the presence or absence of either SAV Ag, oil, CpG/polyI:C or vhsG.

Transcription levels of selected antiviral innate genes, as well as selected B- and T-cell markers were analysed at 12 and 48 hpv in HK (Figure 1) and spleen (Figure 2) for the vaccine formulations in relation to the control group. It is worth noting the often high standard deviations underlining the highly individual immunological response, which is common in fish. Tables with average Cq-values are included as supplementary material.

Results showed that IFNa1, IFNγ and two antiviral proteins induced by IFNs (Mx and Vig-1) were strongly induced by the CpG/polyI:C-adjuvanted treatments, which confirms earlier findings [30]. In detail, for the CpG/polyI:C-adjuvanted treatments, IFNγ were highly upregulated at both time points in HK (Figure 1A; 60–150-fold) and spleen (Figure 2A; 50–100-fold), which was not seen for any of the other treatments. IFNγ is a cytokine secreted by T- and NK-cells and important for both innate and adaptive immune responses against viral infections. Upregulation of IFNa1 in HK and spleen for CpG/polyI:C-treated groups was significantly higher at 12 hpv than at 48 hpv, when a 10-fold reduction was seen. This is consistent with the fact that Type I IFN is an early induced innate antiviral actor, and in accordance, the antiviral genes, Vig-1 and Mx (induced by IFN Type I), accompanied the IFNa1-induction and were still highly expressed at 48 hpv in the CpG/polyI:C-treated groups with approximately a 40-fold induction in spleen and 100-fold for HK. Generally, the antiviral immune gene expression pattern seen for SAV Ag alone and for the formulations without CpG/polyI:C was moderate in both organs (zero- to two-fold in general). In HK, SAV Ag alone induced a low, but significant, upregulation of IFNγ, Vig-1 and Mx at 48 hpv compared to control fish. Antiviral gene responses in HK for vhsG immunized fish were in general moderate and not significantly higher than those in the other groups, except for the CpG/polyI:C-treated groups. Compared to SAV Ag CpG/polyI:C, the immune gene expression patterns for SAV Ag CpG/polyI:C vhsG were similar in both organs, except a slight, but significant, upregulation of IFNa1 and Vig-1 in HK at 48 hpv and downregulation of IFNγ in spleen at 48 hpv. In spleen at 48 hpv, IFNa1 was significantly upregulated in all vhsG-treated groups compared to SAV Ag alone (0.8- to 2.3-fold). Besides that, the expression pattern levels in spleen were similar to those in HK.

PAX5, soluble (s) and membrane-bound (m) IgM were chosen as B-cell markers. PAX5 belongs to the family of paired box transcription factors and is present during early B-cell development, but must be repressed for plasma cell differentiation to take place [42]. Expression of mIgM often occurs in parallel to PAX5, and the ratio of mIgM *vs*. sIgM is important in relation to B-cell development. Early and developing B-cells have higher levels of mIgM and no or low sIgM levels, whereas a shift in their ratio indicates the presence of antibody producing B-cell populations [43]. In general, induction of all three B-cell markers was low at both time points, and the expression pattern varied for the two immune organs. At 12 hpv, a slightly higher expression of PAX5 and mIgM was measured in HK (Figure 1B) for vhsG-treated groups with fold inductions from two to four compared to the other treatments (approximately 1.5 to 2.5). PAX5 was the only B-cell marker induced at 12 hpv in HK of fish treated with SAV Ag, SAV Ag oil or SAV Ag CpG/polyI:C. At 48 hpv, a slight increase of mIgM from 0.8- to 1.5-fold was present for these latter groups, which could indicate a later onset of early B-cell development in groups not receiving vhsG. In spleen (Figure 2B), no clear expression pattern could be seen, and the B-cell markers were hardly detectable until 48 hpv, except for the SAV Ag CpG/polyI:C-treated group, where both PAX5 (2.4-fold) and mIgM (2.3-fold) were induced at 12 hpv. At 48 hpv, the expression of PAX5 was reduced, and both sIgM and mIgM were induced for fish treated with SAV Ag CpG/polyI:C and SAV Ag CpG/polyI:C vhsG.

Co-receptors for T-helper cells (CD4) and cytotoxic T-cells (CD8) were used as T-cell markers to further study early adaptive responses. Atlantic salmon contain two shorter CD4 domain molecules (CD4-2a and -2b) in addition to the four classical (CD4-1) domains [44]. To determine which T-cell subsets were activated, both CD4-1 and CD4-2a, in addition to CD8α transcript levels were measured in HK (Figure 1C) and spleen (Figure 2C) at 12 and 48 hpv. As for B-cell markers, the general induction of T-cell markers in both organs was low and, with a few exceptions, their mRNA levels differed little between treatments and sampling time points. As the supplementary data show, the basal levels of CD4-2a (average Cq-values of 29.7 and 27.5 at both time points for HK and spleen, respectively) were higher in both tissues and at both time points compared to CD4-1 (average Cq-values of 33.6 and 32.4 at both time points for HK and spleen, respectively), although CD4-2a levels were not affected in the two organs at either time point. CD4-1 and CD8α were the most highly upregulated in HK at 12 hpv in groups co-injected with vhsG, ~2.3- to four-fold for both markers, which declined to an average of 1.3-fold for both CD4-1 and CD8α over the next 36 h. At 48 hpv, CD4-1 and CD8α levels in HK were significantly higher for the SAV Ag alone treatment (2.6 and 2.2, respectively) compared to the other treatments (0.8–1.4 for CD4-1 and approximately 1.25 for CD8α). In spleen, the overall expression levels of T-cell markers were very low at both 12 and 48 hpv, except at 48 hpv for SAV Ag CpG/polyI:C vhsG and vhsG alone, where both CD4 markers were upregulated. 

### 3.2. SAV Neutralizing Ab Responses

**Figure 3:**
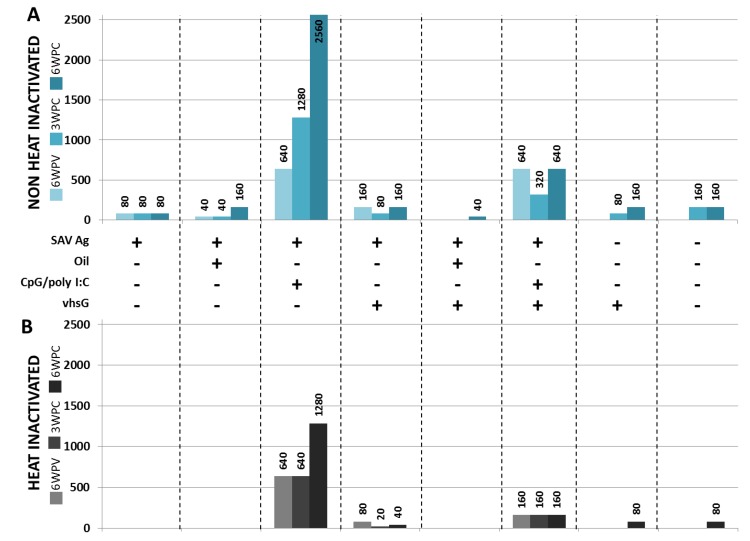
Vaccine-induced anti-SAV neutralizing titres from not heat inactivated (**A**) and heat inactivated (**B**) sera, collected at 6 wpv, 3 wpc and 6 wpc (the colour codes for each time point are next to the *y*-axis). Titres representing a 50% reduction, calculated as described in the Materials and Methods, are shown above the histogram corresponding to each treatment. + or −, respectively, indicates the presence or absence of either SAV Ag, oil, CpG/polyI:C or vhsG.

**Figure 4:**
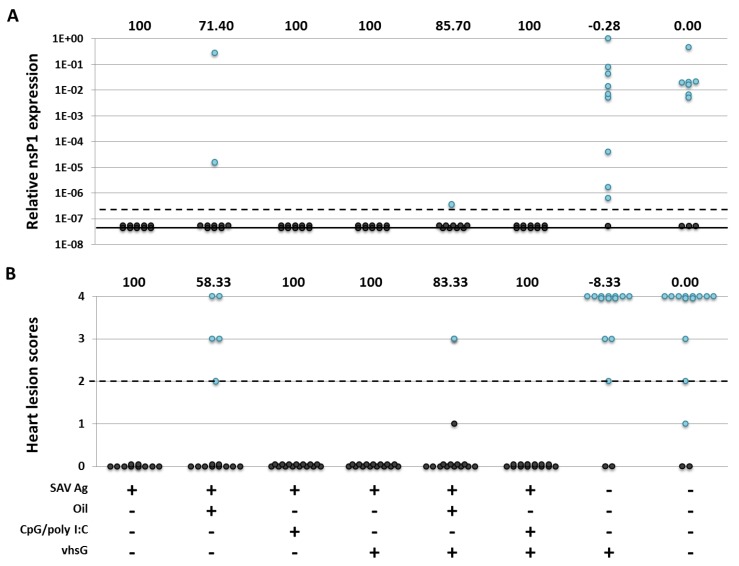
Protection against pancreas disease (PD) in vaccinated and control groups based on (**A**) the reduction of the prevalence of viremia and (**B**) the reduction in severity of SAV-specific heart lesions. (**A**) Relative SAV nsP1 expression at 3 wpc measured by SAV nsP1 RT-qPCR for each treatment group. Individual Cq-values were transformed to RelCq numbers, as described in the Materials and Methods. One (1.0 × 10^0^) indicates the highest presence of nsP1 transcripts. Sera below the dotted line (cut off; 3.0 × 10^−7^) were considered negative, and sera below the solid line had undetected Cq-values. Relative SAV nsP1 values are presented as black (<3.0 × 10^−7^) and blue (≥3.0 × 10^−7^) dots. (**B**) The distribution of individual heart lesion scores assessed by histology at 6 wpc for each treatment group. A score of ≥2 was set as the cut off (indicated by the dotted line). Individual heart lesion scores are presented as black (<2) and blue (≥2) dots. Relative percent protection score (RPPsc.) values corresponding to each group are shown above Graphs A and B. + or −, respectively, indicate the presence or absence of either the SAV Ag, oil, CpG/poly I:C or vhsG DNA construct.

The presence of anti-SAV neutralizing responses in sera was measured at 6 wpv and at three and 6 wpc by a viral neutralization assay. It is well known that heat sensitive factors in serum may augment the neutralizing activity of Ab [45], and therefore, neutralizing activity was measured both with HI and NHI sera. For the NHI sera (Figure 3A), detectable neutralizing antibody titres (nAbTs) were present from 6 wpv for all groups, except groups treated with SAV Ag oil vhsG, vhsG alone or saline. The highest nAbTs were found in the SAV Ag CpG/polyI:C-treated group with titres of 640 before challenge that rose to 1280 and further to 2560 at three and 6 wpc, respectively. Fish treated with SAV Ag CpG/polyI:C that also received the vhsG i.m. injection showed the second highest nAbTs of 640 and 320 at 6 wpv and 3 wpc, respectively and 640 at 6 wpc. While all groups receiving a SAV Ag formulation, except the SAV Ag oil vhsG group, mounted a detectable neutralizing response before challenge, groups receiving either vhsG alone or saline had detectable nAbTs first after challenge. For vhsG alone and saline, the nAbTs are most likely induced upon exposure to the challenge virus, with titres of 80 (3 wpc) and 160 (6 wpc) for vhsG and 160 for saline at both three and 6 wpc. For HI sera, nAbTs were only present in three groups pre-challenge (Figure 3B). CpG/polyI:C-adjuvanted treatments provided the highest responses, with nAbTs ranging from 640 at 6 wpv to 1,280 at 6 wpc for SAV Ag CpG/polyI:C, and for SAV Ag CpG/polyI:C vhsG, the generation of nAbs was consistent with a titre of 160 at all sampling points. No positive sera were found among the fish injected with SAV Ag, SAV Ag oil or SAV Ag oil vhsG, while SAV Ag vhsG had detectable nAb responses both pre- and post-challenge. Fish treated with vhsG and saline, where >80% of the fish in both groups had positive SAV-specific heart lesions at 6 wpc (Figure 4B), showed detectable nAbTs at 6 wpc (80 for both treatments).

### 3.3. Protection

Six weeks after vaccination, all fish were challenged by cohabitation with an SAV Subtype 3 isolate, and vaccine-induced protection was measured at three, five and 6 wpc (Figure 4). No mortality was observed after challenge. At 3 wpc, during the viraemic phase [46], viral RNA from sera were isolated, and SAV nsP1 transcript levels were detected by RT-qPCR. At 3 wpc, 70% (seven out of 10 fish) of the saline-treated fish had SAV positive sera, thus indicating a successful challenge (Figure 4A). Four of the six water-formulated SAV Ag treatments, namely SAV Ag, SAV Ag CpG/polyI:C, SAV Ag vhsG and SAV Ag CpG/polyI:C vhsG, provided full protection (RPPsc. = 100%) against SAV at 3 wpc, with non-detectable Cq-values. For the two oil-formulated groups, SAV Ag oil and SAV Ag oil vhsG, 20% and 10% of the fish had nsP1 positive sera, leading to RPPsc. of 71.4% and 85.7%, respectively. Furthermore, based on the prevalence of viremia determined by nsP1 RT-qPCR, there was a significant difference between all water- and oil-formulated SAV Ag treatments compared to saline-treated fish, except for SAV Ag oil (RPP.sc. = 71%). The prevalence of nsP1 positive fish in the group treated with vhsG alone (nine out of 10 serum positive fish) was significantly higher than the prevalence in the other treatment groups, except the saline group. Protection for the vhsG alone treatment was less than for the saline injected fish with an RPPsc. of −0.28%.

Heart tissue was sampled at five and 6 wpc to evaluate the severity of SAV-induced heart lesions by histological scoring. Protection based on the reduction of the severity of SAV-induced heart lesions was comparable to the protection shown through the reduction of the prevalence of viremia at 3 wpc for all treatments. Here, only histology data for 6 wpc are presented (Figure 4B), given that the prevalence of fish with SAV-specific heart lesions in the control group was highest at 6 wpc (80% *vs*. 60% at 5 wpc), where 10 out of 12 individuals had severe heart lesions. Further, at 6 wpc, lesions in the group vaccinated with SAV Ag oil vhsG were reduced, and only one out of 15 fish showed moderate lesions (RPPsc. 83.3%). The group treated with SAV Ag oil had an RPPsc. of 58.3%, and five fish showed mild to severe lesions, compared to an RPPsc. of 100% at 5 wpc (not shown), with only two fish showing minimal heart lesions. However, there was no significant difference in protection for that treatment between both time points.

## 4. Discussion

How innate and adaptive immunity interact upon vaccination affects the outcome of protection. Especially, TLRs, included in the PRR-family, have emerged as key components of the innate immune system, activating signals critically involved in the initiation and maintenance of adaptive responses. Thus, TLR stimuli can be exploited as powerful adjuvants to elicit both primary and anamnestic immune responses. In light of this, our group used a combination of a selected CpG ODN [47] and a synthetic dsRNA (polyI:C), known to activate PRR-family members specialized in viral and nucleic acid detection (TLR-3, -9, -22 and RIG I), as a model to study the mechanisms of adjuvant action in bony fish. We showed that this TLR-ligand adjuvant combination induced a strong modulation of core response genes and increased levels of nAbs [30].

Here, to follow up and to extend this model, the CpG/poly I:C combo was i.p. injected alone or combined with an i.m. injection of the VHSV G DNA genetic adjuvant. Montanide ISA763A was included as a control adjuvant, i.p. injected in combination with the genetic adjuvant. This was to determine if any enhanced protective responses were induced, since oil-adjuvanted formulations previously had provided a lower protection, most likely due to depot effects [29,30]. An inactivated SAV whole-virus formulation was used as the test Ag and injected i.p. with the same suboptimal dose for all formulations, hypothesized to give about 70% protection based on previous unpublished results. However, in the present SAV challenge, fish injected with SAV Ag alone were fully protected (RPPsc. of 100%), thus preventing the detection of differences in protection between non-oil-based treatments. SAV Ag formulated with Montanide ISA763A oil had a lower efficacy (RPPsc. of 58.3%–85.7%) and produced lower nAb titres (25%–70% reduction) compared to the formulations receiving the equivalent water-based formulation, a difference that could be explained by the slower release of the Ag from the vaccine depot for the oil-adjuvanted formulations.

Correlating to earlier reports, the present CpG/polyI:C formulation induced a distinctively higher upregulation of early innate antiviral transcripts (IFNa1, Vig-1 and Mx) and also higher titres of nAbs compared to all other treatments [26,29,30]. nAbs are thought to be the primary correlate of protection against SAV [7,9,10], and the results suggest that an efficient clearance of virus mediated by Ab is possible. Interestingly, IFNγ expression levels were highly elevated in the CpG/poly I:C-adjuvanted groups. IFNγ, the hallmark of Th1 responses in mammals, is produced mainly by activated T-cells, NK cells and NKT cells; however, other cells, including macrophages/DCs, as well as B-cells are known to express IFNγ upon CpG-stimulation [48]. In support of this, a recent paper showed that salmon MHCII-positive mononuclear phagocytes, as well as B-cells and putative T-cells showed highly upregulated IFNγ transcript levels upon CpG-stimulation [49]. Since IFNγ has been shown to upregulate TLR9 expression in salmon leukocytes [22], the increased levels of this cytokine may represent a positive feed-back loop, where the secreted IFNγ upregulates the TLR9 receptor, and thereby, the cell’s responsiveness to its own agonist are increased.

In higher vertebrates, B-cells activated via PRRs and/or by Ag cross-linking of the B-cell receptor rapidly respond, proliferate and differentiate into IgM secreting short-lived plasma cells (SLPC). T-cell help is needed to induce long-term B-cell memory, represented by memory B-cells or long-lived plasma-cells (LLPC) [50,51]. Bony fish Ab secreting cells (ASCs) are known to possess comparable B-cell subpopulations [52]; however, the understanding of the mechanisms by which these subpopulations are produced and distributed is scarce. Since germinal centres and antibody isotype shifting are not found in bony fish, the classical T-helper function in fish can be questioned. It is thus possible that CpG ODNs (through PRRs) can activate salmon B-cells directly, so that once activated, they start to proliferate and mature into ASCs. This would allow polyclonal activation of the entire B-cell pool, which has been demonstrated for mammalian B-cells [53,54]. Salmon B-cells express TLR9 and are responsive to their own agonist, CpG DNA [55]. Here, the CpG/poly I:C-adjuvanted vaccines were injected into the peritoneum, and recent studies have revealed that IgM-positive cells dominate the peritoneal cavity of unstimulated rainbow trout [56]. Upon inflammatory stimulation, these IgM positive cells were found at high levels 72 h post-injection. Since bony fish B-cells are phagocytic [57], it is possible that the vaccine can be engulfed by B-cells in the peritoneum and directly activated by CpG ODNs to differentiate into ASCs or, alternatively, migrate into secondary immunological organs, such as HK or spleen, and at those sites become ASC. This interesting possibility should be further elucidated in future studies.

Moreover, Desvignes *et al.* [9] have previously suggested that the complement in general might aid in the clearance of SAV. Other studies have since then shown that salmonid Abs are dependent on complement activity to neutralize VSHV and IHNV [58,59], which both are enveloped viruses. SAV is also an enveloped virus; hence, it is possible that complement factors are involved in SAV neutralization, as well. A previous study indeed showed induced levels of complement component C4 (classical pathway) in salmon treated either with saline or with CpG/polyI:C after SAV challenge [29]. In this study, a major fraction of the nAb titres were heat sensitive, underlining the involvement of the complement system in the clearance of SAV. The addition of naive salmon serum as the complement source was evaluated in the virus neutralization assay using HI sera samples. Interestingly, for the sera with low titres, *i.e*., SAV Ag CpG/poly I:C vhsG, the fresh complement increased nAbs, while for the high titre group (SAV Ag CpG/poly I:C), there was no increase in nAbs (results not shown). A possible explanation for the variation in the results for the different groups could be that in the groups with very high nAbT, the neutralizing activity by the Abs *per se* is very efficient, and therefore, the complement does not provide any additional effect. In the groups with lower nAbT, the results indicate that combining the complement and Abs increases neutralization, supporting a role of complement-mediated neutralization, for example, by the classical complement pathway. Further studies aimed at elucidating the significance of the complement for the neutralizing responses need to be addressed.

B- and T-cell markers were, in general, not, or very modestly, induced for all formulations tested, which could be due to the early sampling time points. The results show that fish treated with SAV Ag CpG/polyI:C had a slight, but notable, increase of PAX5 transcripts levels at 12 hpv in both HK and spleen. In spleen, PAX5 induction occurred in parallel to that of mIgM at 12 hpv, while at 48 hpv, PAX5 expression was reduced and mIgM remained stable. This indicates that an early B-cell development takes place [43]. In rainbow trout, activated B-cells differentiate into plasmablasts and plasma cells (PC), both in HK and spleen, and are distributed through the blood system to peripheral tissues [43,52], where hydroxyurea-resistant PC can migrate back to the anterior kidney and may persist there as LLPCs. In mammals, LLPCs are generated by migration to a supportive niche in the bone marrow [50]. The ability of LLPCs to produce Ab for months to years without the stimulating Ag relies on specialized cues. One suggested cue is Type I IFN, which, when injected as an adjuvant in mice, has been shown to induce both short- (10 dpv) and long-lived (26 wpv) Ab production [60]. It has been suggested that the signals induced by Type I IFN affect either migration to survival niches or differentiation of PC [61]. It may well be possible that CpG/polyI:C through its strong induction of Type I IFN could enhance the generation of a, if present, similar long-lived Ab production in salmon, and our intent is to further investigate this aspect.

The immunological mechanisms behind the full protection provided by SAV Ag alone have been reasoned to depend on a T-cell independent (TI) nAb response, as that formulation did not induced an antiviral immune gene response at 5 dpv [30]. Here, a moderate induction of IFNa1, Mx, Vig-1 and IFNγ was evident in HK of fish treated with SAV Ag alone at 12 hpv. Inactivated viral Ags based on other enveloped viruses have been shown to trigger IFN responses [62,63]. Here, the actual mechanism responsible for the IFN responses observed *in vivo* with SAV Ag alone is unknown; nonetheless, by analogy with other alphaviruses, it can be hypothesized that the inactivated SAV particles present in the Ag preparation might induce the Type I IFN response seen, possibly through binding to the mannose receptor of *N*-glycans present on SAV E2, as observed for other alphaviruses [64] and other enveloped viruses [63]. The effect that the moderate induction of innate responses has on cellular immunity later in the course of the challenge is complex to interpret, owing to the limited knowledge on cellular immunity against viral infections in fish. Interestingly, NHI sera from fish treated with SAV Ag alone displayed nAb activity, while there was no detectable nAbT in HI sera for the group receiving SAV Ag alone. This variation between NHI and HI sera emphasizes the importance of the complement in the clearance of SAV and also the suggested ability of CpG to activate B-cells polyclonal through TLR 9, based on the high generation of heat-stable nAbs evident here for SAV Ag CpG/polyI:C compared to SAV Ag alone.

To our knowledge, very few studies have been performed on teleost to investigate the immunostimulatory and protective effects of an i.p. vaccine injected simultaneously with a DNA-based adjuvant injected i.m. The efficacy of the DNA vaccines based on the glycoprotein G from VHSV and IHNV are well-documented; these vaccines have been shown to induce a long-lasting protection against these viruses [65,66], and a commercial IHNV-G DNA vaccine is approved for use in Canadian aquaculture [67]. An early induced cross-protection after VHSV G DNA vaccination has been seen following infection with nodavirus in turbot (*Scophthalmus maximus*) [68] and with the heterologous Novirhabdovirus IHNV in rainbow trout. This study also showed that the DNA expressed G protein does not confer protection against bacterial diseases [69], hence emphasizing that the early non-specific protection provided is purely antiviral. In one study on rainbow trout [70], two or four CpG motifs were incorporated into the plasmid backbone along with VHSV G open reading frame, and this modified DNA vaccine gave significantly higher immune responses (Mx and IFNγ) and a significantly higher production of anti VHSV nAb compared to when plasmid without CpG motifs were administered. Based on these findings, it was hypothesized that the G protein might be able to contribute with a non-specific antiviral protection also against an SAV infection. As evident here, VHSV G glycoprotein DNA vaccination showed no additive effects on early protective responses against SAV. Furthermore, co-injecting vhsG with CpG/poly I:C or oil-adjuvanted SAV Ag neither caused additive nor synergistic effects on the immune gene expression. Muscle samples (*n* = 8) harvested at 48 hpv from vhsG co-injected groups were analysed for vhsG-transcripts, and the levels varied between individual fish, where some individuals had undetectable vhsG mRNA levels (results not shown). The low transcription of vhsG in muscle and the weak effect on innate immune gene expression could be explained by the relative early sampling time points. Indeed, studies on rainbow trout have shown that it can take as long as 14 dpv (earlier samplings at two and 7 dpv did not show any expression) before ISGs are significantly upregulated in spleen [70], and similar results in the liver of rainbow trout [71] and kidney of Japanese flounder (*Paralichthys olivaceus*) [72] have also been described. Protective immune responses observed after challenge in G DNA-vaccinated rainbow trout have been related to increased Mx expression and other ISGs [31,35,73], preceded by the upregulation of IFN Type I and II [35,74]. This early non-specific protection has been followed by a more specific long-lasting anti-viral response, based on both humoral (nAb) and cellular protective mechanisms (MHCII, T-cells) [73,75,76]. Interestingly, both B- and T-cell markers analysed for all vhsG formulations in this study had the highest expression compared to all other groups and were most distinct at 12 hpv in HK. This could indicate that the glycoprotein G has been expressed, and the high standard deviations indicate a high variation between individuals related to either immune gene expression profile and/or of vhsG expression.

In regards to humoral immunity, diverse responses were seen in the different groups i.m. injected with vhsG. While the SAV Ag oil vhsG treatment showed reduced nAbs titres compared to SAV Ag oil, the water-based SAV Ag formulation co-injected with vhsG had higher titres than SAV Ag alone at both 6 wpv and 6 wpc. Finally, a reduction in neutralizing responses was present when vhsG was co-injected with the SAV Ag CpG/polyI:C formulation, and the NHI nAbTs were reduced by half or more at all three time points compared to SAV Ag CpG/polyI:C. These data show that vhsG induced a slightly higher production of nAbs for the SAV Ag-treated fish co-injected with vhsG, and it also suggests the presence of factor(s) that cause a reduction in humoral responses when CpG/polyI:C-adjuvanted SAV Ag is co-injected with vhsG. For both mammals and teleosts, it has been shown that polyvalent vaccination often negatively affects the generation of specific Ab [77,78,79]. Skinner *et al.* [78] have shown that concurrent i.p. vaccination of a polyvalent oil vaccine with i.m. injection of a rhabdovirus-specific DNA vaccine delayed seroconversion of IHNV-specific nAb compared to DNA vaccination alone. The negative effects on nAbT when SAV Ag CpG/poly I:C was co-injected with the vhsG vaccine can thus have several explanations, such as antigenic competition or Ag immunodominance. However, without the possibility of analysing the presence of vhsG-specific Ab (due to the limited amount of sera remaining), neither explanation can be claimed for certain.

## 5. Conclusions

To summarize, the vaccine based on a suboptimal dose of inactivated SAV whole virus Ag formulated with CpG/polyI:C induced the highest nAb responses, followed by the combined treatment of SAV Ag vhsG and, finally, the oil-adjuvanted SAV Ag formulation. The expression of several innate antiviral immune genes showed consistently elevated levels in the groups injected with CpG/polyI:C compared to the other adjuvants tested. B- and T-cell markers were, in general, not, or very modestly, induced for all formulations tested. For groups receiving the vhsG DNA vaccine, no antiviral immune gene expression was detected at these early time points, but as indicated by Martinez-Alonso *et al*. [70], a later induction could be possible. Pre-challenge humoral responses for SAV Ag co-injected with vhsG had slightly higher levels of both heat-sensitive and heat-stable neutralizing factors compared to SAV Ag alone, suggesting a very moderate adjuvant effect of vhsG. No enhancement of nAbs responses was evident when co-injecting vhsG with the TLR-ligand-adjuvanted SAV Ag formulation. Instead, a negative influence was observed, which may result from Ag competition.

Overall, these data show that CpG/polyI:C is a potent TLR-ligand combo, which could be used in future salmonid vaccination strategies against SAV. Further work should be aimed at investigating the duration of the efficacy of these TLR-ligands as adjuvants, while at the same time facilitating their administration. One potential strategy may be to protect the TLR-ligand/Ag formulation from degradation by encapsulating them in protective vehicles, such as nanoparticles, as recently described in [80]. Considering the potency of the TLR-ligand combo tested here, one can anticipate that CpG/polyI:C used as an adjuvant in an SAV Ag formulation could provide an Ag dose-sparing effect.
